# The Utility of the YLS/CMI-SV for Assessing Youth Offenders in Singapore

**DOI:** 10.1177/0093854814537626

**Published:** 2014-12

**Authors:** Chi Meng Chu, Hui Yu, Yirong Lee, Gerald Zeng

**Affiliations:** Ministry of Social and Family Development; De Montfort University; Ministry of Social and Family Development

**Keywords:** criminogenic needs, cross-cultural, predictive validity, recidivism, risk assessment, screening

## Abstract

The Youth Level of Service/Case Management Inventory–Screening Version (YLS/CMI-SV) is designed to provide a preliminary estimate of the level of risk for antisocial behaviors as well as an indication of areas for intervention in youth offenders. This study examined the predictive validity of the YLS/CMI-SV for violent, nonviolent, and general recidivism in a sample of 3,264 youth offenders within a Singaporean context (*M_follow-up_* = 1,764.5 days; *SD_follow-up_* = 521.5). Cox regression and Receiver Operating Characteristic analyses revealed that the YLS/CMI-SV is significantly predictive of general, violent, and nonviolent recidivism for the male youth offenders, but there were mixed results for the female youth offenders. Overall, these results indicated that the YLS/CMI-SV is a useful measure for assessing the levels of risk for male youth offenders, and more investigation is needed to determine the suitability of the YLS/CMI-SV for the female youth offenders. Its implications for clinical practice and policy are discussed.

It is widely accepted that youth offending is an important social issue, and there is a need to assess risk factors and consider treatment needs for those youth who are involved in offending behaviors for the purpose of rehabilitating them (Hoge & Andrews, 1996; McGrath & Thompson, 2012). Historically, the assessment of reoffending risk and identification of intervention needs of offenders have been based on unstructured clinical judgment, but the advent of structured risk assessment measures has led to assessment practices that are more systematic and empirically based. This has, in turn, provided more valid and consistent assessments of risk factors and treatment needs (Hoge, 2002).

## Risk-Needs-Responsivity (RNR) Framework

The RNR framework (Andrews & Bonta, 2010) is a model that emphasizes the usage of structured assessment and decision-making with regard to offender assessment and rehabilitation. According to the RNR framework, effective offender rehabilitation requires the accurate classification of the offender’s level of risk and needs. Accurate identification and classification of risk and needs will allow clinicians to make informed decisions about the levels of supervision, as well as the type and intensity of the treatment interventions that should be provided. The RNR framework is underpinned by general personality and cognitive social learning (GPCSL) theoretical perspectives, which posits that the criminal conduct of individuals is heavily influenced by the “Big Four” variables of antisocial cognition, past antisocial behavior, antisocial personality patterns, and antisocial associates. In addition to these four variables, difficulties pertaining to substance use, family and marital relationships, education and/or employment, and leisure activities make up the “Central Eight” variables that are implicated in criminal offending behavior according to the GPCSL perspectives (Andrews & Bonta, 2010).

The RNR framework states that the intensity of intervention should be matched to the level of risk that the offender poses; moreover, intervention should target those criminogenic needs that are functionally related to criminal behavior, and that the style and mode of intervention should match the offender’s abilities and learning style (Andrews & Bonta, 2010). Pertaining to offender rehabilitation, interventions that adhere to the RNR principles have been associated with significant reductions in recidivism rates, whereas those interventions that fail to adhere to the RNR principles are not linked to improved outcomes in terms of recidivism rates (Andrews & Dowden, 2005). Meta-analyses of risk factors and criminogenic needs with various offender groups have elucidated our understanding of the major, moderate, and minor risk factors for reoffending (e.g., Bonta, Law, & Hanson, 1998; Gendreau, Little, & Goggin, 1996; Hanson & Morton-Bourgon, 2005; Lipsey & Derzon, 1998), and have provided support for “Big Four” as well as “Central Eight” risk and need factors (see Andrews & Bonta, 2010; McGuire, 2004, for a review).

## Youth Level of Service Measures

The Youth Level of Service/Case Management Inventory (YLS/CMI) is one of the most widely used structured risk and need assessment measures across many jurisdictions; for example, Canada, the United States of America, Australia, Japan, Singapore, and the United Kingdom (Chu, Lee, et. al., 2013; McGrath & Thompson, 2012; Onifade et al., 2008; Rennie & Dolan, 2010; Schmidt, Campbell, & Houlding, 2011; Takahashi, Mori, & Kroner, 2013). The YLS/CMI comprises 42 items that relate to the “Central Eight” risk and need domains. The YLS/CMI–Screening Version (YLS/CMI-SV; Hoge & Andrews, 2009) is an eight-item abbreviated measure of the YLS/CMI; the YLS/CMI-SV was trialed in Canada during the early 2000s, but was only recently published and made commercially available. It is designed to provide an initial screening of risk and need levels of youth offenders for the purpose of determining whether a more thorough risk and need assessment is necessary. As with the YLS/CMI, completion of the YLS/CMI-SV within a clinical/forensic setting would normally be based on a file review and interviews with the youth, parent(s), and other collateral sources. Unsurprisingly, the eight items of the YLS/CMI-SV correspond to the eight risk/need domains of the YLS/CMI, with the static factor being *History of Conduct Disorder* and the dynamic factors being *Current School/Employment Problems, Some Criminal Friends, Alcohol/Drug Problems, Leisure/Recreation; Personality/Behavior, Family Circumstances/Parenting*, as well as *Attitudes/Orientation*.

With regard to the YLS measures’ predictive validity, Schwalbe (2007) found a mean-weighted area under curve (AUC) of .64 for recidivism (which includes rearrests and/or readjudication) based on a meta-analytic review of 11 YLS studies. The AUC of the Receiver Operating Characteristics Curve, which range from 0 (perfect negative prediction) to 1.0 (perfect positive prediction), are often considered indices of overall predictive validity. As a general rule for practice, AUCs greater than .54, .63, and .71 are regarded as small, moderate, and large effects, respectively (Rice & Harris, 2005). In an overlapping but larger meta-analytic review of 19 studies, Olver, Stockdale, and Wormith (2009) found that the mean-weighted correlation between YLS total scores and general recidivism was .32, and also showed that the YLS measures had lower predictive validity for general recidivism (which includes any form of violent, nonviolent, and sexual offenses committed during the follow-up period) when they were used in other Western contexts outside of Canada (mean-weighted correlation of .26 vs. .35), which could be due to cross-jurisdiction differences. In follow-up studies, correlation coefficients (*r*) that are greater than .10, .24, and .37 are considered small, moderate, and large effect sizes, respectively (Rice & Harris, 2005). As such, the predictive validity of the YLS/CMI total scores for general recidivism can be considered as being moderate in terms of effect size. Notwithstanding the abundance of studies on the YLS/CMI in the Western contexts, there are very few studies on the YLS measures within Asian contexts.

Pertaining to the YLS/CMI-SV, there is only one specific study (to the best of the authors’ knowledge) that preliminarily examined its psychometric properties (Van de Ven, 2004) and none within the Asian context. In particular, the YLS/CMI-SV Total Score was significantly correlated with police contacts (*r* = .21), number of new criminal charges (*r* = .39), and number of new criminal convictions (*r* = .20); albeit a couple of effect sizes were relatively modest. In the study, Van de Ven also showed that the YLS/CMI-SV Total Score was significantly correlated with the YLS/CMI Total Score (*r* = .69), the *Child Behavior Checklist*^1^ Total Score (Achenbach & Rescorla, 2001; *r* = .51), the *Youth Self-Report*^2^ Total Score (Achenbach & Rescorla, 2001; *r* = .46), the *Criminal Sentiments Scale*^3^ Total Score (Shields & Simourd, 1991; *r* = .46), the *How I Think Questionnaire*^4^ Total Score (Gibbs, Barriga, & Potter, 2001; *r* = 49), and the *Jesness Inventory Asocial Index*^5^ (Jesness, 1998; *r* = .50). Furthermore, the YLS/CMI-SV items were significantly correlated with the corresponding YLS/CMI subscales: *Family Circumstances/Parenting* (*r* = .42), *Education/Employment* (*r* = .70), *Peers* (*r* = .41), *Substance Abuse* (*r* = .78), *Leisure/Recreation* (*r* = .39), *Personality/Behavior* (*r* = .64), and *Attitudes/Orientation* (*r* = .25). Overall, it appears that the YLS/CMI-SV’s preliminary psychometric properties are promising and that its Total Score is correlated with emotional and behavioral difficulties, procriminal attitudes, cognitive distortions, as well as antisocial personality dimensions.

## The RNR Framework and Usage of the YLS Measures in Singapore

Singapore is an independent island-state in South East Asia with a total population of 5.4 million (Singapore Department of Statistics, 2013). Crime rates are generally low in Singapore, and youth arrests account for about 10% of all arrests (Singapore Police Force, 2013). Singapore is a member of the Commonwealth of Nations, and many statutes are based on English common law (e.g., the Criminal Procedure Code, 2012). However, there are some statutes that are based on legislation from other jurisdictions; for example, the Penal Code (2008) is based on the Indian Penal Code, which was (nonetheless) first formulated by the English in 1800s. As such, there are similarities in the way that offenses are defined in Singapore when compared with the above-mentioned countries, but the exact language of the laws might vary somewhat. In particular, cultures and societies often define what attitudes and behaviors are considered “normal” and “deviant.” Notwithstanding that there is some degree of agreement across cultures about what constitutes offending behavior, development of deviant attitudes and behaviors can differ due to cultural norms, gender roles, morals, religion, taboos, and expectations. Importantly, there could be cross-cultural differences as to how individuals cope, self-regulate, or even report crime, so it is possible that the motivation, risk factors, and pathways for offending may differ cross-culturally—making it necessary to examine the empirical evidence whenever there is any adaptation of assessment and intervention frameworks (that are developed for the Western contexts) into non-Western contexts, as well as the accompanying measures. For example, measures might need new norms or cutoffs that would suit the new context due to the aforementioned factors.

In the early 2000s, the RNR framework was introduced in Singapore to provide a theoretical and empirical-based approach to conduct offender assessment and rehabilitation, with the YLS/CMI chosen as the primary risk assessment measure to assess the risk and needs of youth offenders (Chua, Chu, Yim, Chong, & Teoh, in press). Although the YLS/CMI was chosen as the primary risk assessment measure for the juvenile justice agencies in Singapore, the widespread adoption of the YLS/CMI across the various agencies for risk assessment purposes only occurred between 2008 and 2012. As such, coding criteria and cutoffs for risk categories for the Singaporean samples were subsequently developed for assessing the local youth offenders and in accordance with the local legislation and procedures. Following the adoption of the YLS/CMI as the primary risk assessment measure, several youth justice agencies are also considering the use of the YLS/CMI-SV as a screening measure for risk of general recidivism. If implemented, the YLS/CMI-SV could be used as the screening measure for youth offenders and when their risk levels are indicated as being *Moderate* or *High*, further assessment using the YLS/CMI would be warranted.

## The Present Study

Considering that there is currently limited empirical knowledge pertaining to the YLS/CMI-SV’s predictive validity for recidivistic outcomes, two objectives were outlined for the present study. First, the present study aimed to evaluate the predictive validity of the YLS/CMI-SV for predicting different recidivistic outcomes within an Asian context (considering the possible cross-cultural differences pertaining to motivation, risk factors, pathways, and possible reporting of crimes), and whether there were differences in the predictive validity for recidivistic outcomes when the YLS/CMI-SV was rated for male and female youth offenders. The second objective was to explore the relationship between the specific risk factors as assessed on the YLS/CMI-SV and recidivistic outcomes.

## Method

### Sample

The sample consisted of 3,264 youth (aged 12-18 years) who were referred to the Probation Services Branch of the Ministry of Social and Family Development (Singapore) between January 2004 and December 2008, and were placed on community supervision. Their mean age was 15.42 years (*SD* = 1.17), and there were 2,951 (90.41%) males and 313 (9.59%) females in our sample. With regard to ethnicity, 1,750 (53.62%) were Chinese, 302 (9.25%) were Indian, 1,042 (31.92%) were Malay, and 170 (5.21%) were of other ethnic backgrounds. The current sample represents 96.85% (3,264/3,370) of the youth offenders who were placed on community supervision during this period; the remaining could not be coded as a result of missing information or file retrieval difficulties.

Pertaining to offense characteristics for the present sample, 63 (1.93%) had a prior offense history. In addition, the mean number of index offenses committed was 2.61 (*SD* = 2.82, range = 1-40); 1,030 (31.56%) had committed violent index offense(s) (e.g., physical assault, rioting, murder, and robbery), 69 (2.11%) had committed sexual index offense(s) (e.g., indecent exposure, molestation, peeping, rape, and sodomy), and 2,563 (78.52%) had committed nonviolent nonsexual index offense(s) (e.g., theft, fraud, burglary, drug use, and drug trafficking).

### Youth Risk Assessment Measures

#### YLS/CMI-SV

The YLS/CMI-SV (Hoge & Andrews, 2009) contains eight items corresponding to the eight risk/need domains of the YLS/CMI: (a) *History of Conduct Disorder*, (b) *Current School or Employment Problems*, (c) *Some Criminal Friends*, (d) *Alcohol/Drug Problems*, (e) *Leisure/Recreation*, (f) *Personality/Behavior*, (g) *Family Circumstances/Parenting*, as well as (h) *Attitudes/Orientation*. For the YLS/CMI-SV items, a score of “1” indicates the presence of risk factor whereas a score of “0” indicates that absence of risk factor. As such, the total score for the YLS/CMI-SV (range = 0-8) can be obtained by adding the individual item scores. The score cutoffs of the risk bins for the youth offenders are 0 to 2 (*Low*), 3 to 5 (*Moderate*), and 6 to 8 (*High*).

#### YLS/CMI 2.0

The YLS/CMI (Hoge & Andrews, 2011) is a structured assessment instrument designed to facilitate the effective intervention and rehabilitation of youth who have committed criminal offenses (aged 12-18 years) by assessing their risk level, criminogenic needs, and strengths. It consists of 42 items (scored as either *present* or *absent*) that are divided into eight subscales (*Prior or Current Offenses/Dispositions, Family Circumstances/Parenting, Education/Employment, Peer Relations, Substance Abuse, Leisure/Recreation, Personality/Behavior, and Attitudes/Orientation*). The item scores (i.e., rated 1 = *presence of risk factor* or 0 = *absence of risk factor*) can be aggregated to obtain a total risk/needs score. In addition to the eight subscales, the YLS/CMI also consists of items that pertain to noncriminogenic needs and responsivity factors, which can be rated as present or absent. It should be noted that the YLS/CMI 2.0 coding descriptions for some items were adapted with consultation to suit the Singaporean context (R. Hoge, personal communication, March 6, 2013), but the items remained essentially the same. The YLS/CMI was included in this study solely to examine how well the YLS/CMI-SV correlates with it; the findings for the YLS/CMI have been elaborated in another study (see Chu, Lee, et al., 2013).

The guidelines for the administration of the YLS/CMI and YLS/CMI-SV recommend that either a mental health professional or a probation officer complete the measure using information obtained from (a) interviews with the youth, (b) reviews of clinical records, and (c) other collateral sources (Hoge & Andrews, 2009, 2011). However, it is an accepted practice for the YLS measures to be rated using archival records (e.g., case notes and clinical reports) for research purposes (e.g., Chu, Ng, Fong, & Teoh, 2012; Takahashi et al., 2013; Viljoen, Elkovitch, Scalora, & Ullman, 2009).

### Procedure

Ethical approval for this retrospective study was granted by the Ministry of Social and Family Development of Singapore. Case files were obtained from the Probation Services Branch, and reviewed by two psychologists, one probation officer, as well as five research assistants who were trained in the use of YLS measures via attending a 3-day YLS training workshop, readings, and scoring three case studies for practice. The clinical files contained (a) psychological reports prepared by psychologists at Clinical and Forensic Psychology Branch , (b) pre-sentence reports prepared by probation officers, (c) charge sheets, (d) statement of facts, (e) any previous assessment and treatment reports, as well as (f) school reports. Psychological and pre-sentence reports contained specific information pertaining to the youth’s upbringing, interaction with peers and authorities, general and academic functioning, values, family and school environments, as well as information relating to the youth’s offending, treatment, management/supervision, and responsivity issues. In terms of examining the inter-rater reliability, the raters had separately coded a randomly selected sample of 31 files, and the intra-class correlation coefficients for single raters (using absolute agreement definition; ICCs) were .63 (*good*) and .51 (*fair*) for the YLS/CMI and YLS/CMI-SV, respectively (see Cicchetti, 1994, for a classification of ICCs).

### Recidivism Data

Official recidivism data were only obtained following the completion of coding of all other variables; the end of the follow-up period was April 20, 2011. The mean follow-up period was 1,764.5 days (*SD* = 521.5, range = 840-2,666). During this follow-up period, all official records, such as breaches to the conditions of probation, or any type of reoffense that was subsequently charged, were coded. Apropos to the definitions, *general recidivism* refers to any presence of sexual, violent, or nonviolent offenses that were committed following the initial court order, breaches of court orders, or any combination of the aforementioned outcomes. *Violent recidivism* refers to the violent offenses (e.g., physical assault, rioting, murder, and robbery) that were committed following the initial court order, whereas *nonviolent recidivism* refers to the nonviolent nonsexual offenses (e.g., theft, fraud, burglary, drug use, and drug trafficking) that were committed after the initial court order. In addition, *sexual recidivism* refers to sexual offenses (e.g., indecent exposure, molestation, peeping, rape, and sodomy) that were committed after the initial court order. Table 1 shows the breakdown of our sample in terms of committing new offenses during the follow-up period and across gender. The YLS/CMI-SV’s predictive validity for *sexual recidivism* was not examined in the present study because the YLS/CMI has limited utility in assessing youth who sexually offended within the Singapore context (Chu et al., 2012), and also there were only 17 youth (0.52%) who committed sexual offenses during the follow-up period in our sample. Similarly, the YLS/CMI-SV’s predictive validity for *violent recidivism* was not examined for the female subgroup because only 3 of the female youth (1.0%) had committed violent offenses during the follow-up period.

**Table 1:** Breakdown of the Type of Recidivism for the Overall Sample and Subgroups

	Total	Male	Female
	*(N = 3,264)*	*(n = 2,951)*	*(n = 313)*
Recidivism During Follow-Up Period	*N (%)*	*n (%)*	*n (%)*
General	1,228 (37.6)	1,133 (38.4)	95 (30.4)
Nonviolent	1,095 (33.6)	1,001 (33.9)	94 (30.0)
Violent	336 (10.3)	333 (11.3)	3 (1.0)
Sexual	17 (0.5)	17 (0.6)	0 (0)

### Plan of Analyses

Descriptive statistics were used to characterize the sample, whereas correlational analyses were also conducted to examine the relationship between continuous data and the recidivistic outcomes (dichotomous data). Cox regression and Receiver Operating Characteristics (ROC) analyses were conducted to examine the predictive validity of the YLS/CMI-SV Total and Item scores. Cox regression analyses were also conducted to examine whether the YLS/CMI-SV Overall Risk Classifications were predictive of recidivistic outcomes while accounting for differences in follow-up period. In addition, ROC analyses were conducted to examine the predictive validity of the YLS/CMI-SV Total scores for the entire follow-up period, as well as 1-, 3-, and 5-year follow-up periods (see, for example, Chu, Thomas, Ogloff, & Daffern, 2013; Gray, Taylor, & Snowden, 2008). The ROC, which generates an AUC, is a commonly used technique for examining the predictive validity of risk assessment measures, and it is less dependent on the base rates of reoffending than traditional measures of predictive accuracy (Douglas & Webster, 1999). To compare the AUCs of ROC Curves for different follow-up periods, *z*-tests for independent groups (Hanley & McNeil, 1982) were used. Critical ratio *z* is defined as $z=(A_{1}-A_{2})/\sqrt{SE_{1}^{2}+SE_{2}^{2}}$, where A_1_ and A_2_ were the AUCs and the *SE*s were the corresponding standard errors for the AUCs; and *z* values of ≥ |1.96| were taken as evidence that the “true” areas under the ROC Curves were different. Benjamini and Hochberg False Discovery Rate (FDR) corrections (Benjamini & Hochberg, 1995) were conducted to minimize the likelihood of Type I error that might arise from computing multiple comparisons. Analyses were conducted with SPSS 19.0.

## Results

### Univariate Analyses

Means and standard deviations for each of the YLS/CMI-SV items and their correlation with the corresponding YLS/CMI subscales are presented in Table 2. The correlation between the scores of each YLS/CMI-SV item and its corresponding YLS/CMI subscale are reported in Table 3. Most of the correlations were moderate to high in effect size and statistically significant, except for the *Attitudes/Orientation* domain (*r* = .03, *ns*). The total scores of YLS/CMI and YLS/CMI-SV for the total sample (*r* = .66, *p* < .001), males (*r* = .67, *p* < .001), as well as the females were also significantly correlated (*r* = .54, *p* < .001).

**Table 2:** YLS/CMI-SV Item Means and Standard Deviations

	Total	Male	Female	
	*(N = 3,264)*	*(n = 2,951)*	*(n = 313)*	
YLS/CMI-SV Items	*M (SD)*	*M (SD)*	*M (SD)*	*p*
History of Conduct Problems	0.69 (0.46)	0.69 (0.46)	0.77 (0.42)	**.002**
Current School or Employment Problems	0.68 (0.47)	0.67 (0.47)	0.74 (0.44)	**.011**
Some Criminal Friends	0.91 (0.29)	0.91 (0.28)	0.90 (0.30)	*ns*
Alcohol/Drug Problems	0.09 (0.28)	0.09 (0.28)	0.11 (0.31)	*ns*
Leisure/Recreation	0.89 (0.31)	0.88 (0.32)	0.96 (0.19)	**<.001**
Personality/Behavior	0.47 (0.50)	0.46 (0.50)	0.58 (0.50)	**<.001**
Family Circumstances/Parenting	0.24 (0.43)	0.24 (0.43)	0.22 (0.42)	*ns*
Attitudes/Orientation	0.16 (0.37)	0.16 (0.36)	0.21 (0.41)	**.031**

*Note*. All the *p* values in bold remained significant even after FDR corrections. YLS/CMI-SV = Youth Level of Service/Case Management Inventory–Screening Version; FDR = False Discovery Rate.

**Table 3:** Correlation Between YLS/CMI-SV Items With Corresponding YLS/CMI Subscales

YLS/CMI-SV Items	Correlation With Corresponding YLS/CMI Subscales (*r*)					
	*Total (N = 3,264)*	*p*	*Male (n = 2,951)*	*p*	*Female (n = 313)*	*p*
History of Conduct Problems	.11	**<.001**	.11	**<.001**	.12	**.039**
Current School/Employment Problems	.53	**<.001**	.53	**<.001**	.38	**<.001**
Some Criminal Friends	.44	**<.001**	.44	**<.001**	.45	**<.001**
Alcohol/Drug Problems	.68	**<.001**	.75	**<.001**	.45	**<.001**
Leisure/Recreation	.43	**<.001**	.37	**<.001**	−.01	*ns*
Personality/Behavior	.62	**<.001**	.66	**<.001**	.39	**<.001**
Family Circumstances/Parenting	.24	**<.001**	.23	**<.001**	.17	**.002**
Attitudes/Orientation	.03	*ns*	−.05	**.002**	.24	**<.001**

*Note.* All the *p* values in bold remained significant even after FDR corrections. YLS/CMI-SV = Youth Level of Service/Case Management Inventory–Screening Version; FDR = False Discovery Rate.

**Figure 1:**
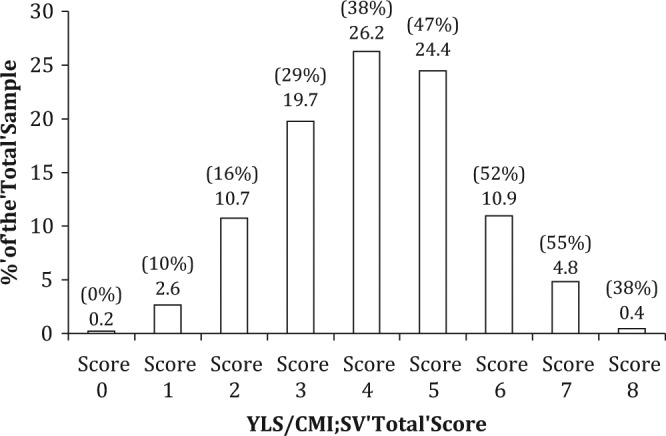
The General Recidivism Rate as a Function of the YLS/CMI-SV Total Score *Note*. The bars represent the proportion of total number of youth offenders (*N* = 3,264) as a function of YLS/CMI-SV Total Scores, with the % indicated on top of each bar. The proportion of youth with the corresponding YLS/CMI-SV Total Score and had reoffended generally is reflected in the parentheses. YLS/CMI-SV = Youth Level of Service/Case Management Inventory–Screening Version

It was noted that females were more likely than males to present with a history of conduct problems, current school or employment problems, a lack of structured leisure/recreational activities, as well as personality/behavioral problems. Apart from *Attitudes/Orientation*, all the YLS/CMI-SV items were significantly correlated with the YLS/CMI subscales. In addition, there was a significant effect for gender in terms of the YLS/CMI-SV Total Scores, *t*(3262) = 4.61, *p* < .001), with females scoring higher when compared with their male counterparts (*M_females_* = 4.48 vs. *M_males_* = 4.09). Importantly, the YLS/CMI-SV Total Score was also significantly correlated to general recidivism during the follow-up period for the overall sample (*r* = .24, *p* < .001), male subgroup (*r* = .26, *p* < .001), and female subgroup (*r* = .15, *p* < .001). Figure 1 shows the proportion of Singaporean youth offenders who engaged in general recidivism during the follow-up period as a function of the YLS/CMI-SV Total Score. It was noted that generally, the higher the YLS/CMI-SV Total Score, the higher the rate of general reoffense.

## ROC Analyses

For the overall sample, the AUCs for predicting general, nonviolent, and violent recidivism using YLS/CMI-SV Total Score were .64 (*SE* = .01, 95% confidence interval [CI] = [.62, .66], *p* < .001), .63 (*SE* = .01, 95% CI = [.61, .65], *p* < .001), and .61 (*SE* = .02, 95% CI = [.58, .64], *p* < .001), respectively. Table 4 shows the AUC values for various recidivistic outcomes for the overall sample and subgroups. The differences between all the AUCs across overall sample and subgroups were nonsignificant in nature, indicating relative stability over 1-, 3-, and 5-year follow-ups.

**Table 4:** AUC Values for the Recidivistic Outcomes Across Entire as Well as Fixed 1-, 3-, and 5-Year Follow-Up Periods

	Overall Sample *(N = 3,264)*			Male Subgroup *(n = 2,951)*			Female Subgroup *(n = 313)*		
Recidivistic Outcomes	*AUC (SE)*	*95% CI*	*p*	*AUC (SE)*	*95% CI*	*p*	*AUC (SE)*	*95% CI*	*p*
General									
Entire follow-up	.64 (.01)	[.62, .66]	**<.001**	.65 (.01)	.63, .67	**<.001**	.59 (.04)	.53, .66	**.008**
1-year follow-up	.64 (.01)	[.62, .67]	**<.001**	.64 (.01)	.62, .67	**<.001**	.66 (.04)	.58, .74	**.001**
3-year follow-up	.65 (.01)	[.63, .67]	**<.001**	.66 (.01)	.64, .68	**<.001**	.62 (.04)	.55, .70	**.002**
5-year follow-up	.65 (.01)	[.62, .68]	**<.001**	.66 (.02)	.63, .69	**<.001**	.65 (.04)	.57, .74	**.001**
Nonviolent									
Entire follow-up	.63 (.01)	[.61, .65]	**<.001**	.64 (.01)	.62, .66	**<.001**	.59 (.04)	.52,.66	**.010**
1-year follow-up	.64 (.01)	[.61, .67]	**<.001**	.64 (.02)	.61, .67	**<.001**	.65 (.04)	.57, .74	**.001**
3-year follow-up	.64 (.01)	[.61, .66]	**<.001**	.64 (.01)	.62, .66	**<.001**	.62 (.04)	.54, .69	**.004**
5-year follow-up	.65 (.01)	[.62, .67]	**<.001**	.65 (.02)	.62, .68	**<.001**	.64 (.04)	.56, .73	**.002**
Violent									
Entire follow-up	.61 (.02)	[.58, .64]	**<.001**	.62 (.02)	.59, .65	**<.001**			
1-year follow-up	.61 (.03)	[.56, .66]	**<.001**	.62 (.03)	.57, .67	**<.001**			
3-year follow-up	.62 (.02)	[.58, .65]	**<.001**	.63 (.02)	.59, .66	**<.001**			
5-year follow-up	.63 (.02)	[.58, .67]	**<.001**	.65 (.02)	.60, .69	**<.001**			

*Note.* All the *p* values in bold remained statistically significant even after FDR corrections. AUC values for violent recidivism were not reported for the female subgroup given that only 3 (1.0%) females committed violent offenses during the follow-up period. The AUC values did not differ significantly across the follow-up periods for the various recidivistic outcomes. AUC = area under curve; FDR = False Discovery Rate.

### Cox Regression Analyses

#### Overall Sample

Cox regression analyses showed that the YLS/CMI-SV Total Score significantly predicted general (β = .29, *SE* = .02, Wald = 209.26, hazard ratio [HR] = 1.33, 95% CI = [1.28, 1.39], *p* < .001), nonviolent (β = .28, *SE* = .02, Wald = 173.73, HR = 1.32, 95% CI = [1.27, 1.37], *p* < .001), and violent (β = .27, *SE* = .04, Wald = 49.90, HR = 1.31, 95% CI = [1.22, 1.41], *p* < .001) recidivism. Table 5 shows the significant predictors of various recidivistic outcomes after accounting for the differences in the follow-up period. Notably, the significant predictors for general and nonviolent recidivism were similar.

**Table 5:** Significant Predictors for General, Nonviolent, and Violent Recidivism in the Overall Sample (Cox Regression; N = 3,264)

	General recidivism					Nonviolent recidivism					Violent recidivism				
YLS/CMI-SV Items	*B*	*SE*	Wald	HR (95% CI)	*p*	*B*	*SE*	Wald	HR (95% CI)	*p*	*B*	*SE*	Wald	HR (95% CI)	*p*
History of Conduct Problems	0.60	0.08	62.39	1.83 [1.58, 2.13]	**<.001**	0.66	0.08	65.61	2.22 [1.82, 2.72]	**<.001**	0.16	0.14	1.42	1.18 [0.90, 1.54]	*ns*
Current School/Employment Problems	0.44	0.07	38.07	1.55 [1.35, 1.78]	**<.001**	0.42	0.08	31.05	1.63 [1.36, 1.95]	**<.001**	0.43	0.14	9.75	1.54 [1.17, 2.02]	**.002**
Some Criminal Friends	−0.03	0.11	0.08	0.78 [0.79, 1.19]	*ns*	−0.06	0.11	0.30	0.94 [0.76, 1.17]	*ns*	0.23	0.22	1.11	1.26 [0.82, 1.93]	*ns*
Alcohol/Drug Problems	0.16	0.09	2.84	1.17 [0.98, 1.41]	*ns*	0.21	0.10	4.12	1.22 [1.01, 1.48]	0.04	0.24	0.17	1.99	1.28 [0.91, 2.21]	*ns*
Leisure/Recreation	0.47	0.12	15.07	1.59 [1.26, 2.02]	**<.001**	0.58	0.06	18.07	1.92 [1.42, 2.59]	**<.001**	0.35	0.23	2.42	1.42 [0.91, 2.21]	*ns*
Personality/Behavior	0.12	0.06	4.36	1.13 [1.01, 1.27]	0.04	0.05	0.06	0.73	1.06 [0.93, 1.19]	*ns*	0.31	0.11	7.31	1.36 [1.09, 1.70]	**.007**
Family Circumstances/Parenting	0.36	0.07	26.63	1.43 [1.25, 1.63]	**<.001**	0.36	0.07	24.25	1.43 [1.24, 1.65]	**<.001**	0.25	0.13	3.51	1.28 [0.99, 1.66]	*ns*
Attitudes/Orientation	0.04	0.08	0.19	1.04 [0.88, 1.21]	*ns*	0.03	0.09	0.15	1.03 [0.87, 1.22]	*ns*	0.20	0.15	1.74	1.22 [0.91, 1.62]	*ns*

*Note.* All the *p*-values in bold remained significant even after FDR corrections. YLS/CMI-SV = Youth Level of Service/Case Management Inventory–Screening Version; HR = hazard ratio; 95% CI = 95% confidence interval.

In addition, Cox regression analyses revealed that the YLS/CMI-SV Overall Risk Classification of *Low, Moderate*, and *High* were significantly different from each other in terms of time to general, nonviolent, and violent recidivism. In terms of *general recidivism*, those youth who were rated as High risk were 5.12 (95% CI = [3.89, 6.71], *p* < .001) and 1.62 (95% CI = [1.37, 1.85], *p* < .001) times as likely to reoffend as compared with those who were rated as Low and Moderate risk, respectively; whereas youth who were rated as Moderate risk of recidivism were 3.17 (95% CI = [2.46, 4.08], *p* < .001) times as likely to reoffend as compared with the Low-risk group. With regard to *nonviolent recidivism*, the High-risk group was 5.03 (95% CI = [3.75, 6.76], *p* < .001) and 1.51 (95% CI = [1.31, 1.75], *p* < .001) times as likely to reoffend as compared with the Low- and Moderate-risk groups. The Moderate-risk group was also 3.33 (95% CI = [2.53, 4.38], *p* < .001) times as likely to reoffend as the Low-risk group. Finally, pertaining to *violent recidivism*, the High-risk group was 4.50 (95% CI = [2.68, 7.58], *p* < .001) and 1.63 (95% CI = [1.27, 2.10], *p* < .001) times as likely to reoffend as compared with the Low- and Moderate-risk groups. The Moderate-risk group was also 2.76 (95% CI = [1.69, 4.52], *p* < .001) times as likely to reoffend as the Low-risk group.

#### Male Subgroup

Cox regression analyses showed that the YLS/CMI-SV Total Score significantly predicted general (β = .30, *SE* = .02, Wald = 212.52, HR = 1.35, 95% CI = [1.29, 1.40], *p* < .001), nonviolent (β*<* = 0.29, *SE* = .02, Wald = 172.87, HR = 1.33, 95% CI = [1.28, 1.39], *p* < .001), and violent (β = 0.30, *SE* = .04, Wald = 59.69, HR = 1.34, 95% CI = [1.25, 1.45], *p* < .001) recidivism for male youth offenders. Table 6 shows the significant predictors of various recidivistic outcomes after accounting for the differences in the follow-up period.

**Table 6:** Significant Predictors for General, Nonviolent, and Violent Recidivism in the Male Subgroup (Cox Regression; n = 2,951)

	General Recidivism					Nonviolent Recidivism					Violent Recidivism				
YLS/CMI-SV Items	*B*	*SE*	Wald	HR (95% CI)	*p*	*B*	*SE*	Wald	HR (95% CI)	*p*	*B*	*SE*	Wald	HR (95% CI)	*p*
History of Conduct Problems	0.57	0.08	52.23	1.77 [1.52, 2.07]	**<.001**	0.63	0.09	54.45	1.88 [1.59, 2.22]	**<.001**	0.21	0.14	2.18	1.23 [0.93, 1.62]	*ns*
Current School/Employment Problems	0.47	0.07	40.23	1.60 [1.38, 1.84]	**<.001**	0.42	0.08	29.43	1.53 [1.31, 1.78]	**<.001**	0.46	0.14	10.66	1.58 [1.20, 2.07]	**.001**
Some Criminal Friends	−0.08	0.11	0.06	0.99 [0.80, 1.23]	*ns*	−0.04	0.12	0.09	0.97 [0.77, 1.22]	*ns*	0.25	0.22	1.22	1.28 [0.83, 1.98]	*ns*
Alcohol/Drug Problems	0.21	0.10	4.70	1.17 [0.98, 1.41]	**.03**	0.26	0.10	6.58	1.30 [1.06, 1.58]	**0.01**	0.27	0.17	2.37	1.31 [0.93, 1.83]	*ns*
Leisure/Recreation	0.47	0.12	15.14	1.60 [1.26, 2.03]	**<.001**	0.58	0.14	18.65	1.79 [1.37, 2.33]	**<.001**	0.41	0.23	3.28	1.51 [0.97, 2.34]	*ns*
Personality/Behavior	0.16	0.06	6.71	1.17 [1.04, 1.32]	**0.01**	0.06	0.07	0.88	1.06 [0.94, 1.21]	*ns*	0.34	0.12	8.89	1.41 [1.13, 1.77]	**.003**
Family Circumstances/Parenting	0.36	0.07	26.63	1.43 [1.25, 1.63]	**<.001**	0.31	0.08	16.45	1.37 [1.17, 1.59]	**<.001**	0.21	0.13	2.40	1.23 [0.95, 1.60]	*ns*
Attitudes/Orientation	0.03	0.09	0.09	1.03 [0.87, 1.22]	*ns*	0.04	0.09	0.18	1.04 [0.87, 1.25]	*ns*	0.25	0.15	2.87	1.29 [0.96, 1.73]	*ns*

*Note.* All the *p*-values in bold remained significant even after FDR corrections. YLS/CMI-SV = Youth Level of Service/Case Management Inventory–Screening Version; HR = hazard ratio; 95% CI = 95% confidence interval.

Moreover, Cox regression analyses further revealed that the YLS/CMI-SV Overall Risk Classification of *Low, Moderate*, and *High* were significantly different from each other in terms of time to general, nonviolent, and violent recidivism. In terms of *general recidivism*, those youth who were rated as High risk were 5.53 (95% CI = [4.17, 7.33], *p* < .001) and 1.65 (95% CI = [1.43, 1.90], *p* < .001) times as likely to reoffend as compared with those who were rated as Low and Moderate risk, respectively; whereas youth who were rated as Moderate risk of recidivism were 3.35 (95% CI = [2.58, 4.35], *p* < .001) times as likely to reoffend as compared with the Low-risk group. With regard to *nonviolent recidivism*, the High-risk group was 5.71 (95% CI = [4.19, 7.77], *p* < .001) and 1.59 (95% CI = [1.37, 1.85], *p* < .001) times as likely to reoffend as compared with the Low- and Moderate-risk groups. The Moderate-risk group was also 3.59 (95% CI = [2.70, 4.78], *p* < .001) times as likely to reoffend as the Low-risk group. Finally, pertaining to *violent recidivism*, the High-risk group was 5.35 (95% CI = [3.14, 9.14], *p* < .001) and 1.73 (95% CI = [1.35, 2.22], *p* < .001) times as likely to reoffend as compared with the Low- and Moderate-risk groups. The Moderate-risk group was also 3.09 (95% CI = [1.86, 5.12], *p* < .001) times as likely to reoffend as the Low-risk group.

#### Female Subgroup

The YLS/CMI-SV Total Score significantly predicted general (β = .23, *SE* = .08, Wald = 7.86, HR = 1.26, 95% CI = [1.07, 1.47], *p* = .005), and nonviolent (β = .22, *SE* = .08, Wald = 7.22, HR = 1.24, 95% CI = [1.06, 1.46], *p* = .007) recidivism for female youth offenders. As mentioned in the “Method” section, the predictive validity of the YLS/CMI-SV for violent recidivism in the female subgroup was not examined due to the very low base rate of violent recidivism (i.e., 1.0%). Table 7 shows the significant predictors of general and nonviolent recidivism after accounting for the differences in the follow-up period; it was noted that only the *History of Conduct Problems* had significantly predicted general and nonviolent recidivism for the female subgroup.

**Table 7:** Significant Predictors for General, Nonviolent, and Violent Recidivism in the Female Subgroup (Cox Regression, n = 313)

	General Recidivism					*Nonviolent Recidivism*				
YLS/CMI-SV Items	*B*	*SE*	Wald	HR (95% CI)	*p*	*B*	*SE*	Wald	HR (95% CI)	*p*
History of Conduct Problems	1.06	0.34	9.86	2.89 [1.49, 5.60]	**.002**	1.05	0.34	9.61	2.85 [1.47, 5.54]	**.002**
Current School/Employment Problems	0.28	0.26	1.12	1.32 [0.79, 2.20]	*ns*	0.25	0.26	0.94	1.29 [0.77, 2.15]	*ns*
Some Criminal Friends	−0.22	0.33	0.45	0.80	*ns*	−0.25	0.33	0.58	0.78	*ns*
			[0.42, 1.52]					[0.41, 1.48]		
Alcohol/Drug Problems	−0.37	0.37	0.97	0.69 [0.33, 1.44]	*ns*	−0.34	0.37	0.81	0.71 [0.34, 1.49]	*ns*
Leisure/Recreation	1.29	1.01	1.63	3.62 [0.50, 26.07]	*ns*	1.27	1.01	1.58	3.54 [0.49, 25.55]	*ns*
Personality/Behavior	−0.03	0.22	0.03	0.97 [0.64, 1.47]	*ns*	−0.03	0.22	0.02	0.97 [0.64, 1.48]	*ns*
Family Circumstances/Parenting	0.26	0.24	1.10	1.29 [0.80, 2.08]	*ns*	0.25	0.25	1.02	1.28 [0.79, 2.07]	*ns*
Attitudes/Orientation	0.19	0.25	0.58	1.21 [0.74, 1.96]	*ns*	0.16	0.25	0.41	1.17 [0.72, 1.91]	*ns*

*Note.* All the *p*-values in bold remained significant even after FDR corrections. YLS/CMI-SV = Youth Level of Service/Case Management Inventory–Screening Version; HR = hazard ratio; 95% CI = 95% confidence interval.

Cox regression analyses were also conducted to examine the utility of the risk bin cutoffs for general and nonviolent recidivism. The results did not suggest that the YLS/CMI-SV Overall Risk Classification of *Low, Moderate*, and *High* were significantly different from each other in terms of time to general recidivism (High-risk vs. Low-risk: HR = 2.57, 95% CI = [0.90, 7.35], *ns*; High-risk vs. Moderate-risk: 1.56, 95% CI = [1.00, 2.45], *ns*; Moderate-risk vs. Low-risk: HR = 1.64, 95% CI = [0.60, 4.52], *ns*) or nonviolent recidivism (High-risk vs. Low-risk: HR = 2.48, 95% CI = [0.87, 7.08], *ns*; High-risk vs. Moderate-risk: 1.56, 95% CI = [1.00, 2.45], *ns*; Moderate-risk vs. Low-risk: HR = 1.55, 95% CI = [0.98, 2.43], *ns*).

## Discussion

This study has examined the predictive validity of the YLS/CMI-SV for general, nonviolent, and violent recidivism using a large sample of Singaporean youth who have offended. In addition, the present study also examined the association between the item and total score of the YLS/CMI-SV with the corresponding total and subscale scores of the YLS/CMI.

### Predictive Validity of the YLS/CMI-SV in a Non-Western Context

#### Overall Sample

The present study suggests that there was predictive validity with regard to the YLS/CMI-SV Total Score. In particular, the general trend was that the higher the total score, the higher the general recidivism rate for the youth offenders (with the exception of YLS/CMI-SV Total Score of 8). This anomaly could be a result of the small proportion of the cases with a total score of 8 (only 13 cases; hence, it is sensitive to fluctuations when described in percentages). Apropos of the relationship between the YLS/CMI-SV items and general recidivism, univariate analyses conducted with the overall sample revealed that the YLS/CMI-SV items were significantly associated with general recidivism with modest effect sizes though *Some Criminal Friends* was not associated with recidivism. Further examination revealed that the *Some Criminal Friends* item did not suitably discriminate between recidivists and nonrecidivists as the majority of the sample had this item endorsed.

In addition, most of the items, apart from *Attitudes/Orientation*, were significantly correlated to the corresponding YLS/CMI subscales when we conducted the analyses using the overall sample. The nonsignificant association between the YLS/CMI and YLS/CMI-SV on the Attitudes/Orientation is contrary to the extant literature on the YLS measures (e.g., Van de Ven, 2004). Further examination of the gender subgroups revealed that the YLS/CMI and YLS/CMI-SV correlated negatively on this item for the male subgroup (albeit a very small effect size), whereas the YLS/CMI and YLS/CMI-SV correlated moderately on this item for the female subgroup. For the male subgroup (with the seemingly anomalous finding), it was possible that the single item examining antisocial attitudes and orientation on the YLS/CMI-SV did not have an adequate coverage of this criminogenic domain as compared with the YLS/CMI, and coding criteria on the YLS/CMI-SV may not have covered the scope examined in the YLS/CMI. However, this remains an empirical question that needs to be further examined and replicated.

Cox regression analyses revealed that four of the eight risk factors were significantly predictive of general and nonviolent recidivism (i.e., *History of Conduct Problems, Current School/Employment, Leisure/Recreation*, and *Family Circumstances/Parenting*), whereas two (i.e., *Current School/Employment* and *Personality/Behavior*) were predictive of violent recidivism after accounting for the influence of other risk factors. In the only other published study on YLS/CMI-SV, Van de Ven (2004) did not obtain recidivism data for the Canadian sample and we are currently unable to make comparisons in terms of the significant risk factors for predicting recidivistic outcomes. However, with regard to the association between the YLS/CMI-SV items and the YLS/CMI subscales, the correlation indices between YLS/CMI-SV items and YLS/CMI subscales as reported in Van de Ven’s study were substantially higher than those found in the present study (e.g., *Family Circumstances/Parenting* [.42 vs. .24], *Current School/Employment* [.70 vs. .53], *Attitudes/Orientation* [.25 vs. .03], and *Alcohol/Drug Problems* [.78 vs. .68]). The correlation indices for *Some Criminal Friends, Personality/Behavior*, and *Leisure/Recreation* were similar for YLS/CMI-SV and YLS/CMI. It was further noted that the YLS/CMI-SV Total Score was significantly correlated with the YLS/CMI Total Score (.66) in this present study and the effect size was very similar to Van de Ven’s (.70).

Although the present study did not examine predictive validity of the YLS/CMI-SV for frequency and nature of the police contacts as well as the number of new charges, it is clear that the YLS/CMI-SV Total Score has significant predictive validity for general, nonviolent, and violent recidivism even after accounting for differences in follow-up periods. In addition, the YLS/CMI-SV Overall Risk Classification has been shown to able to discriminate between the Low-, Moderate-, and High-risk groups effectively for the three recidivistic outcomes, with each group having significant differences in terms of time to reoffend—this suggests that the risk bin cut-offs have sufficient validity. In terms of ROC analyses, the YLS/CMI-SV Total Score has also shown adequate predictive validity for violent, nonviolent, and general recidivism (i.e., AUCs = .61, .63, and .64, respectively) over a mean follow-up period of almost 5 years. These AUCs are generally consistent with those cited in Schwalbe’s (2007) meta-analysis.

#### Male and Female Subgroups

The differences in AUCs for the general and nonviolent recidivism across the various follow-up periods for both subgroups were nonsignificant. However, the effect sizes of the AUCs were largely moderate for both male and female groups (see Table 4). In contrast to the pattern of findings as described in Gray et al. (2008), who found a small decline for a violence risk assessment measure (the Historical, Clinical, and Risk Management – 20 factors [HCR-20]) over time, the predictive validity of the YLS/CMI-SV ratings were maintained over longer follow-up periods. This pattern suggested that the purported dynamic risk factors are relatively stable over the short to medium term. It is imperative that future research studies examine the “shelf-life” of these purported dynamic risk factors.

With regard to gender differences, risk and need profiles for our sample indicated a different pattern compared with those of Canadian youth offenders (Hoge & Andrews, 2011). Specifically, Singaporean youth female offenders, as compared with their male counterparts, scored higher for *History of Conduct Problems* (i.e., the only static/historical factor), and several dynamic risk factors including those relating to *Current School/Employment Problems, Leisure/Recreation, Personality/Behavior*, as well as *Attitude/Orientation* (see Table 1). However, male youth offenders in Canada scored higher than their female counterparts on seven out of the eight domains (with the exception of Alcohol/Drug Problems). It appears that the female youth offenders in Singapore have a higher level of criminogenic needs (as measured on the YLS/CMI-SV) when they enter the juvenile justice system as compared with the male youth offenders. Although many of the YLS/CMI-SV items were significantly associated with recidivistic outcomes for the male subgroup, only the *History of Conduct Problems* was significantly associated with recidivistic outcomes for the female subgroup. One possibility could be that there are different pathways for offending across gender, which would be linked to differences in risk factors for offending behavior. Another possibility could be that many “gender-neutral” assessment measures that were developed for males had limited relevance for females; thus, some risk factors and needs that are most relevant to female offenders might have been omitted or ignored (e.g., Taylor & Blanchette, 2009; Salisbury & Van Voorhis, 2009; Van Voorhis, Wright, Salisbury, & Bauman, 2010). Such possibilities should be areas of future empirical inquiry within the Singaporean context.

Finally, there were distinct differences in terms of the YLS/CMI-SV Overall Risk Classifications for both groups. In particular, the YLS/CMI-SV Overall Risk Classifications were suitable for the male subgroup but did not significantly differentiate the Low-, Moderate-, and High-risk offenders in the female subgroup (albeit some comparisons were approaching significance). Thus, the current findings suggest that it is necessary to reexamine the relevance of the risk factors for the female subgroups (or at least the criteria for which each risk factor is rated on), as well as the cutoffs for the Overall Risk Classification using a larger female sample.

### Implications

The YLS/CMI-SV is an abbreviated version of the YLS/CMI, and it comprises eight items that could be easily scored—at a fraction of the time needed to complete the YLS/CMI. As the YLS/CMI-SV is intended as a screening measure, additional assessment using the YLS/CMI would be warranted if the youth is assessed as being at Moderate or High risk of recidivism on the YLS/CMI-SV. The usage of the YLS/CMI-SV as the primary screening measure could help save time that could otherwise be allocated to treatment or case management. In fact, the YLS/CMI-SV can be used in settings whereby a quick and structured screening will be useful to identify those youth offenders with a possibly higher risk of reoffending behavior. Such settings may include institutional or community settings whereby there is a high flow of youth offenders through the system, and limited manpower prevents the practitioners from conducting full-scale risk assessments for every youth offender due to large numbers. The present study has established that the YLS/CMI-SV has adequate predictive validity for recidivistic outcomes (especially for male youth offenders), and the cutoffs for YLS/CMI-SV Overall Risk Classifications are also suitably accurate in terms of discriminating between the Low-, Moderate-, and High-risk male youth offenders within the Singaporean context. Taken together, the YLS/CMI-SV seems suited for use with male youth offenders in Singapore—though the converse cannot be said for the female youth offenders yet. Notably, it provides a standardized screening method to assess risk factors and criminogenic needs in male youth offenders. In Singapore, the YLS/CMI-SV has been recently piloted in youth diversionary initiatives. It will be interesting to examine how useful the measure is when used to assess youth who are diverted from the youth justice system.

The current study also showed that there is a need to consider gender differences in terms of risk assessment/screening. There is also the necessity to examine whether (and to what extent) additional gender-responsive factors contribute to the YLS/CMI-SV in terms of predicting recidivism as well as case management. For example, it is important to consider gender-responsive factors such as victimization and abuse (e.g., Salisbury & Van Voorhis, 2009; Siegel & Williams, 2003), relationship problems (e.g., Robertson & Murachver, 2007; Salisbury & Van Voorhis, 2009), mental health needs (e.g., Benda, 2005; Brown & Motiuk, 2005), substance abuse (e.g., Covington & Bloom, 2007; Neff & Waite, 2007), self-efficacy (e.g., Case & Fasenfest, 2004), and parental issues (Ferraro & Moe, 2003; Ross, Khashu, & Wamsley, 2004) in risk assessment and classification processes. It is insufficient to simply use purported gender-neutral risk assessment measures (that were created for males) to assess females without a thorough examination of the relevance of their risk factors. This is an important issue given that accurate assessment of risk for offending behavior will not only increase clinicians’ confidence in managing the youth offenders, but will also improve public safety and confidence. In addition, accurate identification of risk and needs will help provide fair and equitable management of youth offenders within the system. It is imperative that scholars, practitioners, and policy makers consider this issue of gender-responsive assessments (and ultimately treatment) for female youth offenders. Moreover, policy makers and practitioners need to recognize that a male youth offender is not the same as female youth offender in terms of the risk of general or nonviolent recidivism posed to the community (given the same YLS/CMI-SV score), which ultimately may have implications for supervision and offender rehabilitation practices (e.g., Van Voorhis et al., 2010; Wright, Van Voorhis, Salisbury, & Bauman, 2009).

### Limitations and Future Research

First, we relied on electronic data and archival file data for coding of the risk assessment measures and recidivism follow-up; thus, there would inevitably be an underestimate of recidivism due to further offenses not having been detected. Similarly, the retrospective methodology used in the present study would have also underestimated the presence of dynamic risk factors given that repeated measurements are not possible with our current research design. Second, the number of cases included in the inter-rater reliability check was relatively small and the intra-class correlation coefficient obtained for the YLS/CMI-SV total score was fair (.51); this relatively low inter-rater reliability check may suggest that the ratings for the YLS/CMI-SV could be improved and in doing so, the predictive validity for the YLS/CMI-SV Total Score might be enhanced. Future replications should include a larger sample for inter-rater reliability checks and attempt to obtain higher accuracy indices through training and feedback. Finally, the predictive validity of the measure might be artificially lowered by the clinicians’ initial identification and diffusion of potential criminal offending behavior that might be exhibited during the individuals’ court orders via psychological (e.g., counseling or relaxation), increased supervision, and/or social (e.g., social or sporting activities) interventions. Therefore, it is possible that the predictive accuracy of the risk assessment instrument might be attenuated.

In spite of these limitations, this study used a large sample and appropriate analyses (e.g., ROC analyses and FDR corrections) that are less dependent on reoffending rates and can address Type I error without being overly conservative; henceforth, we can be relatively confident about the results. Importantly, we believe that this is one of the first large-scale studies on the predictive validity of the YLS/CMI-SV with long-term recidivism data. Furthermore, the YLS/CMI-SV can clearly distinguish between the low-, moderate-, and high-risk male offenders, so that clinicians can be afforded important screening information to help them decide whether a more comprehensive risk and need assessment is needed. Overall, this study has yielded much-needed information on the predictive validity and applicability of YLS/CMI-SV for youth offenders in a non-Western context. Future research on youth risk assessment measures should use prospective and repeated measures designs (e.g., using interviews on top of information that is already available in archival records). As aforementioned, it would also be beneficial to investigate the risk factors that are relevant to female offenders and reexamine the items for YLS/CMI-SV for female youth offenders as well as the relevant cutoffs in terms of the Overall Risk Classifications.

## Notes

1. The *Child Behavior Checklist* is a standardized instrument designed to measure child and adolescent competencies, as well as emotional and behavioral problems as reported by parents or guardians.

2. The *Youth Self-Report* is a standardized instrument designed to obtain the youth’s reports of their own competencies and problems.

3. The *Criminal Sentiments Scale* is a 41-item scale that measures attitudes toward the criminal justice system, rationalizations for offenses, and personal evaluative judgments about law violators. Higher scores on the Criminal Sentiments Scale are indicative of greater procriminal attitudes.

4. The *How I Think Questionnaire* is a self-report measure that assesses self-serving cognitive distortion.

5. The *Jesness Inventory* is a self-report measure designed to measure personality dimensions in children and adolescents who are involved in or suspected of delinquency.
